# iTRAQ-based proteomics reveals serum protein changes in hypertensive rats induced by a high-salt diet

**DOI:** 10.17179/excli2020-2740

**Published:** 2020-11-06

**Authors:** Ying Qin, Tao Dong, Wan Jiang, Wen Ding, Tao Zhan, Juan Du, Ren Zhao, Bing Shen, Jiexia Chen

**Affiliations:** 1 School of Basic Medicine Sciences, Anhui Medical University, 81 Meishan Road, Hefei, Anhui, China; 2 Department of Neurology, Drum Tower Hospital, Medical School and The State Key Laboratory of Pharmaceutical Biotechnology, Nanjing University, Nanjing, China; 3 Department of Cardiology, The First Affiliated Hospital of Anhui Medical University, Hefei, Anhui, China; 4 Department of Geriatrics Cardiology, The First Affiliated Hospital of Anhui Medical University, Hefei, Anhui, China

**Keywords:** iTRAQ, LC-MS/MS, proteomics, high-salt diet, hypertension, cardiovascular diseases, endothelial dysfunction

## Abstract

High-salt diets may increase both hypertension and risk of cardiovascular diseases. Although high-salt diets can result in hypertension and impaired vascular function, the molecular mechanisms underlying these dysfunctions are not fully known. Thus, the aims of the present study were to identify key proteins and their signaling pathways and associated molecular mechanisms that may contribute to, as well as be potential biomarkers of, the pathogenesis of hypertension-related cardiovascular diseases. To that end, the present study identified and quantitated serum proteins that were differentially expressed in male rats fed regular chow (n = 4) and those fed a high-salt diet (n = 4) to induce hypertension. The serum was collected from both groups, and the proteins differentially expressed in the serum were identified and quantitated using isobaric tags for relative and absolute quantitation combined with liquid chromatography-tandem mass spectrometry. Of 396 identified proteins, 24 were differentially expressed between the groups: 19 proteins were significantly (*P* < 0.05) upregulated (> 1.2 fold change), and 5 were significantly downregulated (< 0.8 fold change). Gene ontology and Kyoto Encyclopedia of Genes and Genomes enrichment analyses indicated that these differentially expressed proteins may contribute to cardiovascular diseases via the roles they play in endothelial function, vascular remodeling, the coagulation cascade, and the complement system. In addition, phagosome processes and the integrin-associated focal adhesion signaling pathway were determined to be potential underlying molecular mechanisms. The key proteins identified in this study warrant further development as new therapeutic targets or biomarkers of cardiovascular diseases associated with high-salt diet-induced hypertension.

## Introduction

Numerous studies using animal models of hypertension and epidemiological studies have demonstrated that excess sodium intake is an important factor in the occurrence and development of essential hypertension. Approximately 1.65 million people worldwide die of cardiovascular disease every year caused by a high intake of salt (Ibrahim and Damasceno, 2012[15]; Lim et al., 2012[20]; Mozaffarian et al., 2014[23]). Between 1990 and 2013, the global incidence of hemorrhagic and ischemic strokes in individuals between the ages of 20 and 64 years nearly tripled, with about 54 % of strokes attributed to hypertension (He et al., 2013[13]; Krishnamurthi et al., 2015[18]). Many studies have shown that reducing salt intake is associated with lowering blood pressure and with ameliorating cardiovascular disease (Benjamin et al., 2019[1]; He et al., 2020[14]).Therefore, understanding the mechanisms underlying hypertension caused by a high-salt diet is critical for the prevention and treatment of cardiovascular disease. 

Among the mechanisms causing hypertension, salt sensitivity is attracting the most attention. After successfully establishing a rat salt-sensitive hypertension model, Kawasaki et al., and Luft et al., proposed the concept of blood pressure salt sensitivity (Kawasaki et al., 1978[17]; Luft et al., 1977[22]). Current research suggests that a high-salt diet may contribute to high blood pressure and cardiovascular disease by destroying endothelium-dependent relaxation, altering peripheral vascular resistance, increasing sympathetic nervous system arousal, and activating immunity/inflammation (Cao et al., 2017[4]; DuPont et al., 2013[8]; Stocker et al., 2010[32]; Touyz et al., 2018[36]). A high-salt diet mainly stimulates and promotes blood vessel constriction, hypertrophy, fibrosis, and calcification, thereby changing vascular morphology and function; however, the underlying molecular mechanisms are far from elucidated.

Proteins participate in various physiological and pathological processes. In hypertension, specific proteins produced by the cells of tissues may be secreted into the circulation. Therefore, proteomics offers an effective method to detect and quantify those serum proteins and to determine whether they are differentially expressed in hypertension. Isobaric tags for relative and absolute quantitation (iTRAQ) is a protein quantification technology based on tandem mass spectrometry. Compared with other proteomics technologies, it has the advantages of high sensitivity, high resolution, and good reproducibility (Zhang et al., 2018[40]). In the present study, we collected serum from rats fed a high-salt diet or regular chow and used iTRAQ techniques to detect the proteins that were differentially expressed between the two groups. Through bioinformatics analysis of those significantly differentially expressed proteins (DEPs), we sought to elucidate the roles and mechanisms of key proteins and their related signaling pathways in cardiovascular diseases caused by a high-salt diet.

## Materials and Methods

### Animal model and sample collection

All animal experiments complied with the ARRIVE guidelines and were conducted in accordance with the National Institutes of Health *Guide for the Care and Use of Laboratory Animals* (NIH Publications No. 8023, revised 1978). All animal study procedures were approved by Experimental Animal Ethics Committee of Anhui Medical University.

In total, eight male, 6-week-old Sprague Dawley male rats (180 g) were randomly assigned to one of two groups: control or experimental. The control group was provided food containing 0.4 % salt, whereas the experimental group was provided food containing ten times as much salt (4.0 %). Animals in both groups were housed in a controlled environment at 25 °C and were allowed to freely eat and drink water without interference for 4 weeks. After that, the blood pressure (BP) of the rats was measured using a tail-cuff blood pressure meter (Supplementary Table 1). Rats with a systolic BP above 130 mmHg were considered hypertensive. Animals were humanely killed via inhalation of CO_2_ gas. The chest was opened immediately on death, and blood was obtained from the heart by using a syringe. The rat blood samples were placed at room temperature for 1 h and then centrifuged at 3000 × *g* for 15 min. The serum samples were collected and stored at -80 °C for later experimental use.

### Protein preparation and iTRAQ labeling

We used a ProteoMiner Protein Enrichment Kit (Bio-Rad) to remove high-abundance protein in the plasma, and the Bradford method (1976[2]) was used to determine protein concentrations. From each sample, 35 μg of protein was obtained and 5× sample buffer was added to it at a volume ratio of 4:1. A total of nine samples of mixtures (four from the control group and four from the experimental group after removing the highly abundant proteins, and one sample of unprocessed serum from a control rat) were bathed in boiling water for 5 min and centrifuged for 10 min at 14000 × *g*. Each supernatant was collected and electrophoresed using 10 % sodium dodecyl sulfate-polyacrylamide gel electrophoresis (SDS-PAGE) at a constant current of 14 mA for 90 min to separate the proteins and to detect the quality of the removed protein. Dithiothreitol (5 µL of 1 M) was added to 200 µg of each protein solution, and the samples were incubated at 37 °C for 1 h. Then, 20 µL of 1 M iodoacetamide was added, and the samples were mixed and placed in the dark at room temperature for 1 h. The solution was mixed slightly with a pipette and transferred to an ultrafiltration tube. After ultracentrifugation at 14000 × *g* for 20 min at 4 °C, the resulting supernatant was discarded. Next, 100 µL of UA buffer (8 M urea, 100 mM Tris-HCl, pH 8.0) was added to the pellet, the samples were centrifuged at 14000 × *g* for 20 min at 4 °C, and the resulting supernatant was discarded. This step was repeated twice. We then added 100 µL of 0.5 M tetraethylammonium tetra hydroborate to the pellet in the ultrafiltration tube, and the samples were centrifuged under the same conditions. The resulting supernatant was discarded. This step was repeated three times. We replaced the collection tube with a new one and added trypsin to each protein sample according to the (mass) protein to enzyme ratio of 50:1. Enzymatic hydrolysis was allowed for 12-16 h at 37 °C. Finally, we labeled the sample peptides as described in the manual accompanying the iTRAQ Reagent-8Plex Multiplex Kit (AB Sciex). Isobaric tags 113, 114, 115, and 116 were used to label the peptides in the control group, whereas isobaric tags 117, 118, 119, and 121 were used to label peptides in the high-salt diet group.

### High-pH reversed-phase chromatography and LC-MS/MS analysis

The dried iTRAQ-labeled peptide samples were re-dissolved in 100 µL buffer A (2 % ACN, pH 10), vortexed, and centrifuged at 14000 × *g* for 20 min at 4 °C. The resulting supernatant from each sample was loaded onto a C18 column. Peptides were separated by a linear gradient formed from buffer A and buffer B (98 % ACN, pH 10) at a flow rate of 700 µL/min. Beginning at the fifth minute, the eluents were collected in sequence every 1.5 min. After being separated and vacuum-dried, these fractions were used for analysis using liquid chromatography with tandem mass spectrometry (LC-MS/MS). All peptide fractions were dissolved in 5 µL of 0.5 % formic acid (FA) and centrifuged at 14,000 rpm for 10 min at 4 °C. The supernatant was transferred to a loading tube, and the peptides were separated by a linear gradient formed from mobile phase A (0.1 % FA, H_2_O) and mobile phase B (0.08 % FA, 80 % ACN) at a flow rate of 600 nL/min. The separated peptides were placed directly into a mass spectrometer (Q Exactive; Thermo Scientific) for detection.

### Database retrieval and bioinformatics analysis

We used Proteome Discoverer Software to search the uniprot-rattus + norvegicus.20180702_36089.fasta database. Fold change is the liner value of the quantitative ratio in the protein concentration between the two groups of samples. Those peptides in the two groups of our serum samples that were significantly increased or decreased from each other (*P* < 0.05) were selected. The protein expression data of each rat were listed in Supplementary Table 2. Gene ontology (GO) analysis was conducted for those peptides using Metascape, a web-based resource (http://metascape.org) for gene and protein annotation, visualization, and integration discovery (Fang et al., 2019[11]; Soonthornvacharin et al., 2017[31]). Kyoto Encyclopedia of Genes and Genomes (KEGG) pathway analyses were then conducted using the KEGG Orthology-Based Annotation System (KOBAS) online analysis tool (http://kobas.cbi.pku.edu.cn/) (Kanehisa and Goto, 2000[16]) to identify those pathways that were statistically significantly enriched with the peptides of interest.

### Statistical analysis

The two-tailed Mann-Whitney and Fisher exact test were performed with SigmaPlot software. Values are expressed as means ± SEM. A value of *P* < 0.05 was considered statistically significant. The *P* value was not false discovery rate adjusted.

## Results

### Blood pressure change in rats fed a high-salt diet

**Figure 1:**
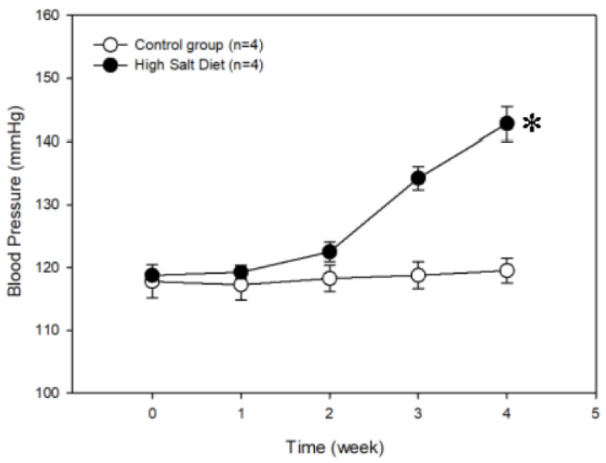
Changes in systolic blood pressure of rats fed a high-salt vs. a regular diet. The values are the means ± SE; n=4 samples in each group. *P < 0.05 for the experimental group vs. control group (regular diet) (two-way ANOVA). The *P* value was not false discovery rate adjusted.

Rats were fed regular-salt chow or a high-salt diet for 4 weeks. Results of their BP measured by a tail-cuff blood pressure meter showed that the systolic BP in the experimental group was higher than 130 mmHg after 3 weeks of eating a high-salt diet, whereas systolic BP in the group fed the normal-salt diet remained within normal reference ranges (Figure 1(Fig. 1), Supplementary Table 1). The difference in BP between the two groups was significantly different at 4 weeks (*P *< 0.05).

### SDS-PAGE

**Figure 2:**
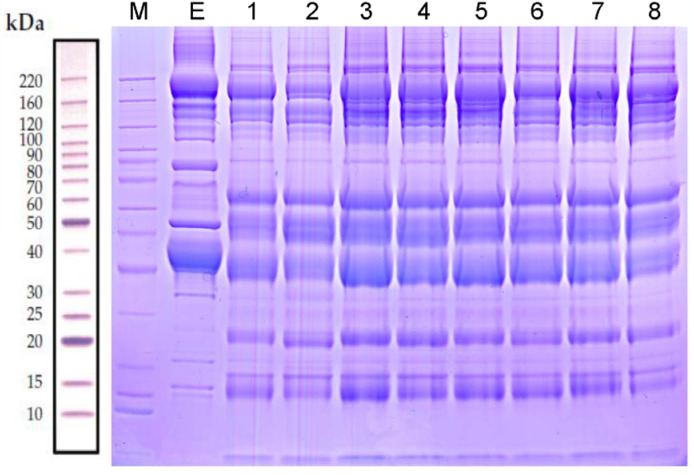
Protein separation and quality control by sodium dodecyl sulfate-polyacrylamide gel electrophoresis. Lane M contains markers. Lane E is the unprocessed serum from a control rat. Lanes 1-4 are serum samples after removing the highly abundant proteins from each of four rats fed the regular control diet. Lanes 5-8 are serum samples after removing the highly abundant proteins from each of the four rats fed a high-salt diet.

Proteins in the serum samples from rats fed a high-salt diet and from the control group were separated by SDS-PAGE. Total proteins in the molecular weight range of 15-220 kDa in the eight samples were effectively separated without protein degradation, indicating that the samples contained proteins of sufficient quality to be used in the subsequent experiments (Figure 2(Fig. 2)).

### LC-MS/MS spectrum analysis and identification of DEPs

**Figure 3:**
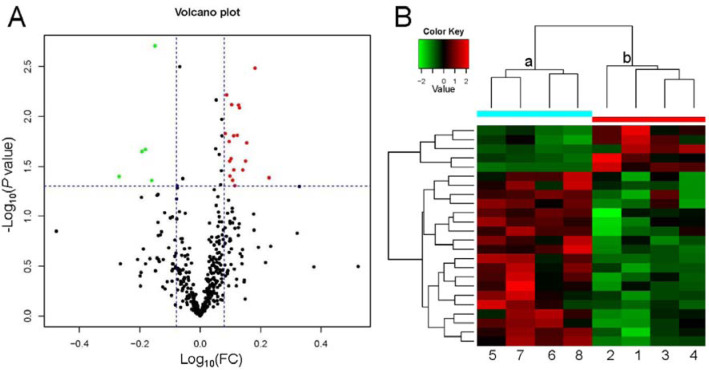
Volcano plot and heatmap of differentially expressed proteins. (A) Volcano plot of differential protein expression. The abscissa is the difference (linear fold change [FC] in protein concentration (logarithmic transformation with base 10), and the ordinate is the statistical significance, that is, the logarithmic transformation with base 10 for the *P* value). Red and green dots in volcano plot indicate proteins with significant differences (green, *P* < 0.05 and FC <0.8; red, *P* < 0.05 and FC >1.2); black dots are proteins without significant change. (B) Heatmap of differential protein expression. Each row represents a protein, each column is a sample/repeat, and each color represents a different quantity of expression (log_10_ for quantitation and median correction). The *P* value was not false discovery rate adjusted.

LC-MS/MS is a powerful tool for identifying proteins in serum. We identified 396 proteins, of which LC-MS/MS analysis identified 24 serum proteins with concentrations significantly different between rats fed a high-salt diet and those fed a regular diet: 19 proteins were upregulated and 5 proteins were downregulated (Table 1(Tab. 1), Figure 3(Fig. 3), Supplementary Table 2). The cluster analysis of these DEPs indicated that the protein expression pattern in the high-salt diet group was significantly different from that of the normal-salt diet group (Figure 3B(Fig. 3)). 

### GO function annotation and enrichment analysis

GO analysis is a useful method and tool in bioinformatics analysis. It includes three categories: cellular component, molecular function, and biological process. GO function annotation indicates the number of DEPs in each of these three categories. GO functional enrichment analysis provides important GO function terms related to the DEPs. Different proteins coordinate with each other to produce biological behavior, and pathway-based analysis helps to further understand these biological functions. Pathway enrichment analysis can determine the most important biochemical metabolic pathways and signal transduction pathways related to DEPs. 

**Figure 4:**
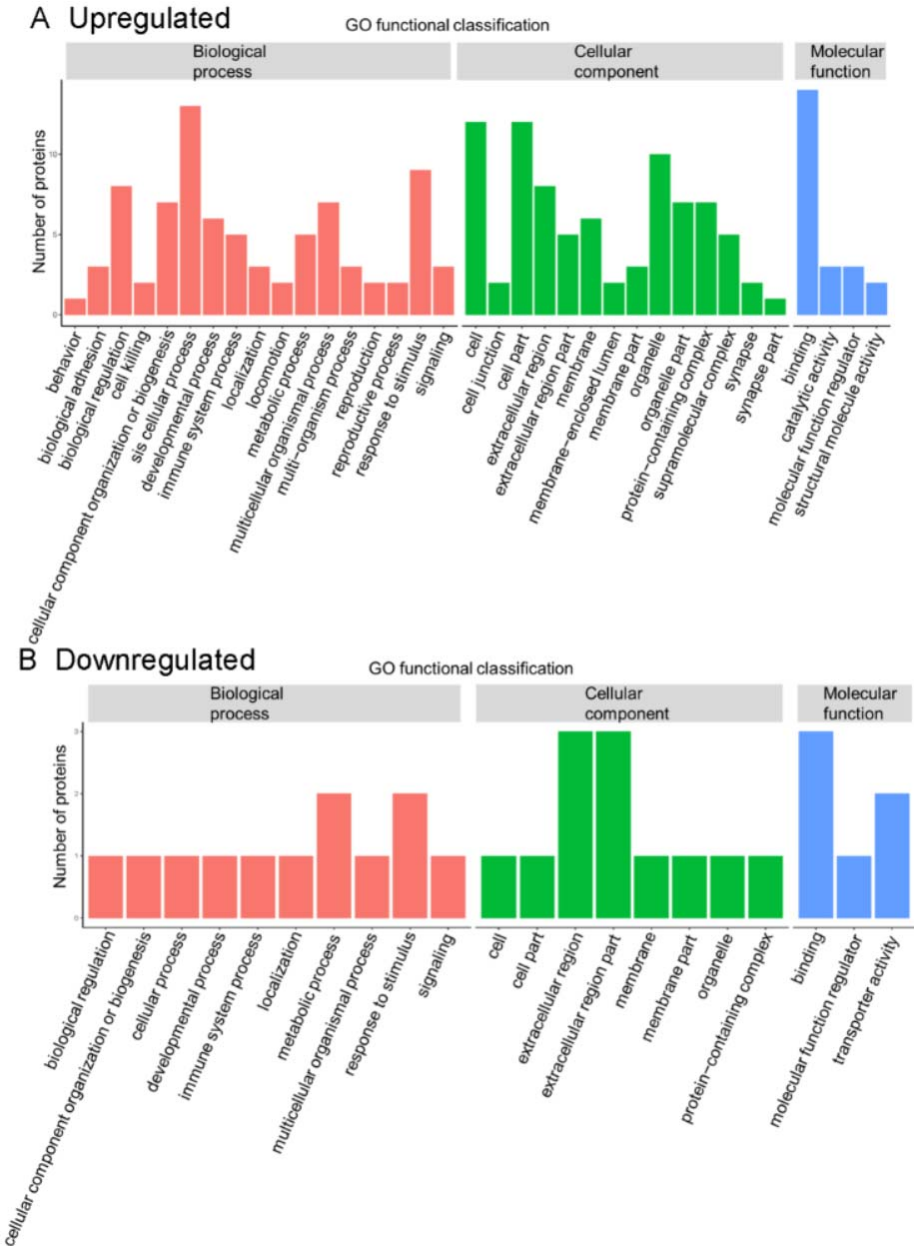
Gene ontology (GO) annotation for functional classification. The abscissa indicates the classification description under each GO classification, and the ordinate represents the number of differentially expressed proteins.

In the present GO function annotation analysis for the classification of biological process, the highest percentage of DEPs were related to the term “cellular process" (n = 13 proteins) (Figure 4(Fig. 4)), with the top three proteins in this cluster being THBS1, FLNA, and CORO1A. For the classification of cellular component, the highest percentage of proteins were related to "cell" (n = 12) or "cell part" (n =12), with the top three upregulated proteins in both two clusters being THBS1, FLNA, and CORO1A. In molecular function classification, the highest percentage of proteins were related to "binding" (n = 14), with the top three upregulated proteins in this cluster being IGD, THBS1, and FLNA.

**Figure 5:**
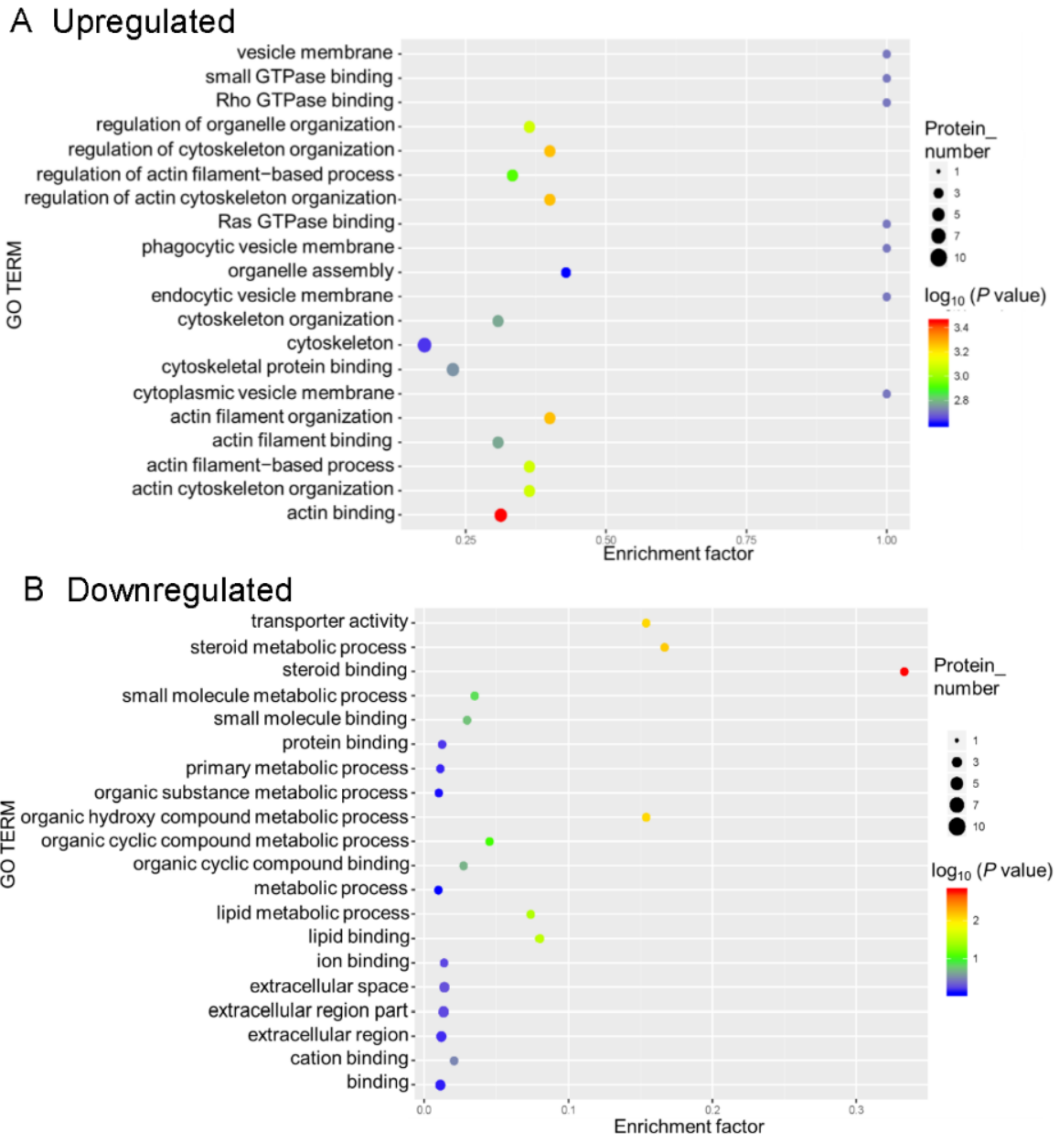
Gene ontology (GO) enrichment for functional terms. The abscissa represents the enrichment factor, that is, the percentage of differentially expressed proteins in the GO classification relative to the identified proteins in that classification. The ordinate indicates the GO term description, that is, the detailed description of the GO classification. The size of the bubble represents the number of proteins in the GO classification. Fisher exact test *P* value: the enrichment test *P* value obtained by using the Fisher exact test; −log_10_(*P* value): the logarithmic conversion of the Fisher exact test *P* value. The *P* value was not false discovery rate adjusted.

For the GO functional enrichment analysis, the enriched upregulated DEPs were mainly for the functional terms cytoskeleton, actin binding, and cytoskeletal protein binding, whereas the enriched downregulated DEPs were primarily related to the functional terms binding and extracellular region part (Figure 5(Fig. 5)).

### KEGG pathway annotation and enrichment

**Figure 6:**
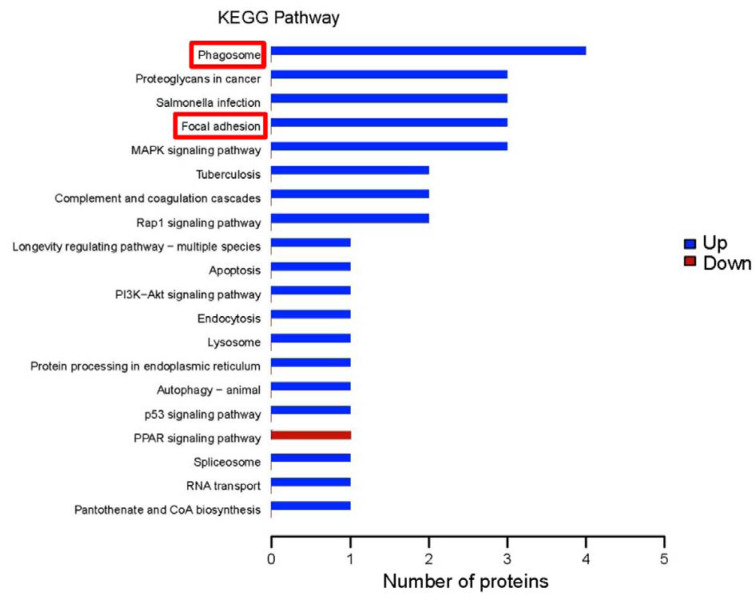
Kyoto Encyclopedia of Genes and Genomes (KEGG) pathway annotations of differentially expressed proteins. The abscissa represents the number of differentially expressed proteins; the ordinate lists the KEGG pathways with differentially expressed proteins. Blue bars indicates upregulated pathways; the red bar, a downregulated pathway. The red-framed pathways indicate underlying molecular mechanisms we predicted.

The KOBAS online analysis tool is used to identify functions related to DEPs and KEGG signaling pathways. The top five annotated KEGG pathways for the upregulated DEPs were phagosome, proteoglycans in cancer, salmonella infection, focal adhesion, and the mitogen-activated protein kinase (MAPK) signaling cascade. The KEGG pathway annotation analysis indicated that the peroxisome proliferator-activated receptor (PPAR) signaling pathway was downregulated (Figure 6(Fig. 6)).

**Figure 7:**
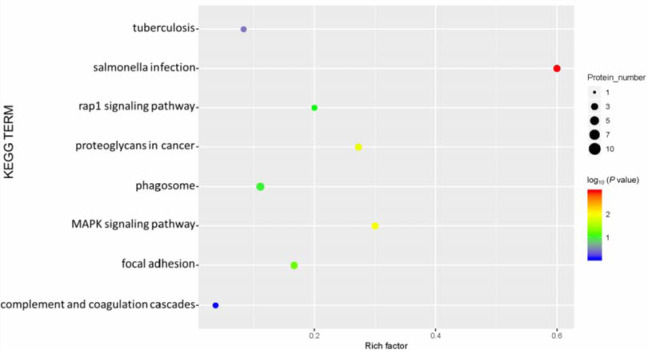
Kyoto Encyclopedia of Genes and Genomes (KEGG) pathway enrichment of differentially expressed proteins. The abscissa represents the enrichment factor: the total proportion of differentially expressed proteins in the KEGG signaling pathway as a multiple of the proportion of identified proteins in the classification. The ordinate lists the KEGG term description. Bubble size indicates the number of differentially expressed proteins in the KEGG pathway; Fisher exact test *P* value: the enrichment test *P* value obtained by using Fisher exact test; −log_10_(*P* value): the logarithmic conversion of Fisher exact test *P* value. The *P* value was not false discovery rate adjusted.

Our KEGG pathway enrichment analysis results showed that phagosome functions, focal adhesion, and the MAPK signaling pathway were the main pathways associated with rats fed a high-salt diet (Figure 7(Fig. 7)).

**Figure 8:**
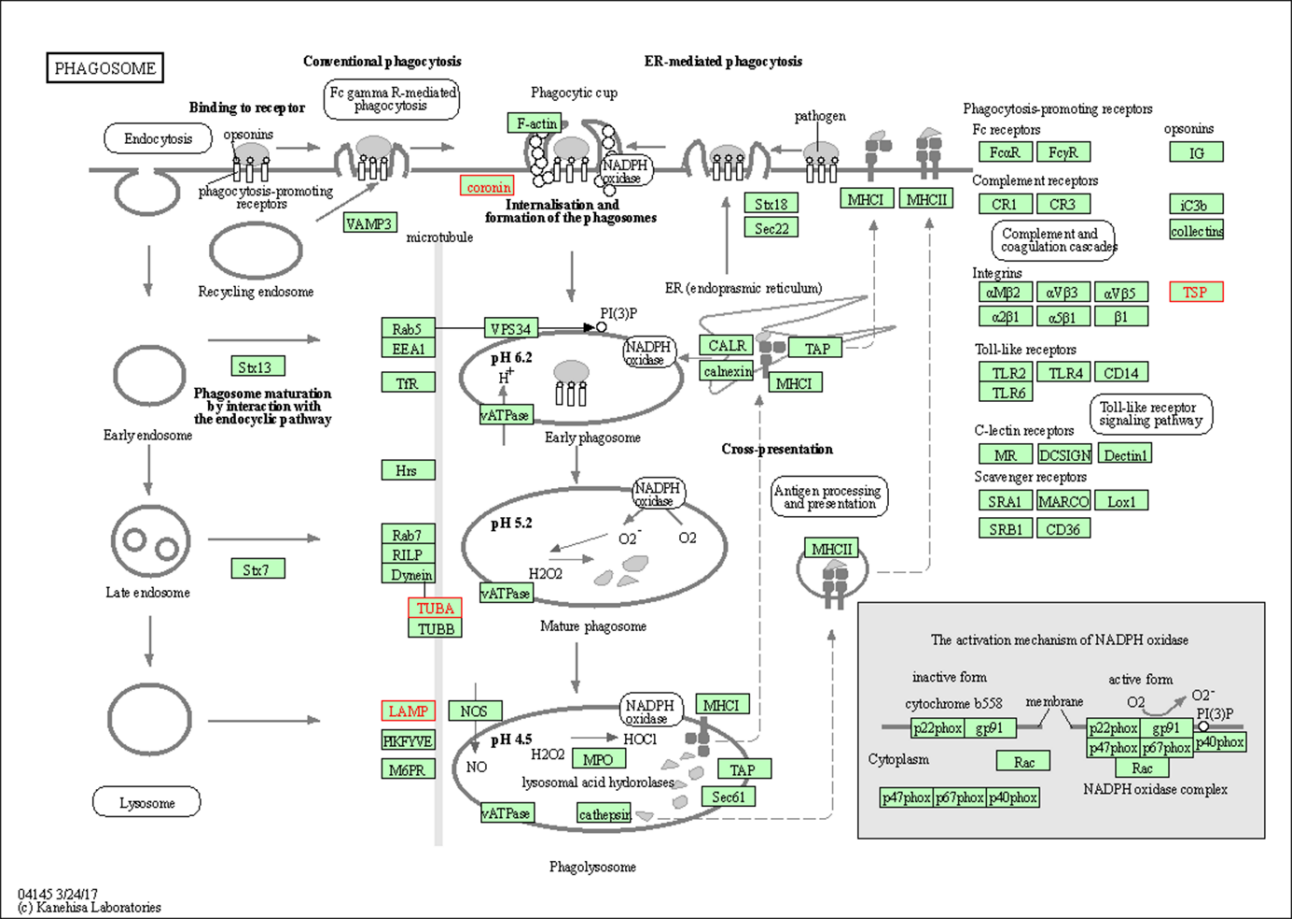
Phagosome dynamics and functions. The upregulated differentially expressed proteins Coronin-1A (coronin), Tubulin Alpha-1A chain (TUBA), lysosome-associated membrane Glycoprotein 1 (LAMP) and Thrombospondin 1 (TSP), all highlighted in a blue frame, participate in phagosome processes.

**Figure 9:**
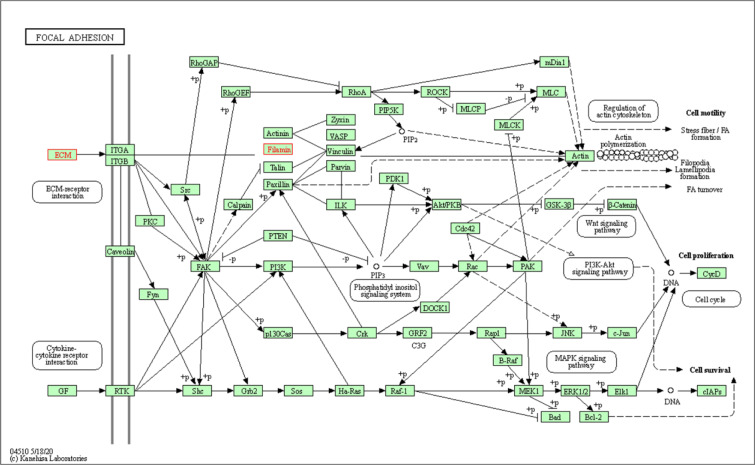
Focal adhesion signaling pathway. The upregulated differentially expressed proteins extracellular matrix (ECM) component and Filamin, both highlighted in a blue frame, participate in the focal adhesion signaling pathway.

Thus, our KEGG pathway annotation and enrichment analyses indicated that phagosomes and focal adhesions were noteworthy. Of the DEGs detected in the present study, those related to phagosomes were THBS1, CORO1A, LAMP1, and TUBA1A (Figure 8(Fig. 8)), and those DEGs related to focal adhesions were THBS1, FLNA, and FLNC (Figure 9(Fig. 9)). 

## Discussion

One-third of the world's population has an excessively high salt intake (Thout et al., 2019[35]), which can cause serious cardiovascular diseases. Genomics and metabolomics studies show that the occurrence of cardiovascular disease is closely related to vascular endothelial disorders (Edwards et al., 2020[9]; Sun et al., 2019[34]). However, the key substances involved in these complex pathological cases are unclear, and it is important to identify those substances. Thus, the present study combined iTRAQ and LC-MS/MS analyses with the goal of identifying the proteins that are differentially expressed between normal-salt and high-salt diets. We identified 396 proteins in the serum of rats, of which 24 were DEGs: 19 proteins were upregulated, and 5 proteins were downregulated. Our KEGG pathway annotation analyses indicated that four upregulated proteins were involved in phagosome processes, and three upregulated proteins were involved in the focal adhesion pathway.

Thrombospondin 1 (THBS1), also known as glycoprotein G, is an adhesive glycoprotein that belongs to the platelet reactive protein family, and it mediates cell-to-cell and cell-to-matrix interactions. Smadja et al., found that an increase in plasma THBS1 levels is positively correlated with cardiovascular disease (Smadja et al., 2011[30]), which is in line with our results. Our study also showed increased levels of Filamin A (FLNA), Filamin C (FLNC), and Myomesin 2 (MYOM2) in the myosin family. In addition, the KEGG pathway analysis indicated that the focal adhesion signaling pathway may play an important role in the development of cardiovascular diseases related to a high-salt diet. THBS1 is generally identified as a main activator of transforming growth factor β (TGF-β). TGF-β comes from the transforming growth factor superfamily and carries out signal transduction by binding to three types of receptors on the cell surface: type I, type II, and type III. Owing to its functional diversity, TGF-β can activate downstream signaling pathways, regulating cell proliferation, differentiation, development, and morphogenesis and acting as an important molecule in the pathogenesis of disease. TGF-β can further activate cells to produce reactive oxygen species by mitochondria or NADPH oxidase 4 (Brown and Griendling, 2015[3]). Reactive oxygen species accumulates the damage of DNA and proteins in the body, which may also be an important mechanism for the injury of blood vessels and body tissues caused by a high-salt diet. TGF-β can also promote fibrosis through a variety of mechanisms (Liu and Desai, 2015[21]), including activation of fibroblasts, stimulation of epithelial and endothelial cell apoptosis, induction of epithelial or endothelial-mesenchymal transition, production of extracellular matrix (ECM) protein, and inhibition of ECM degradation. Sushi, nidogen and EGF-like domain 1 (SNED1) is a large ECM glycoprotein, and our LC-MS/MS results showed that SNED1 is differentially increased in rats fed a high-salt diet. SNED1 has been reported to have substantial effects on tumor progression or metastasis and has been shown to be negatively associated with patient prognosis (Naba et al., 2014[24]). Therefore, THBS1 may be an important molecular component associated with high-salt diet-induced cardiovascular disease by activating its downstream target TGF-β.

Under normal circumstances, the tunica intima of the arterial wall is composed of a single layer of endothelial cells without the presence of smooth muscle cells. However, after vascular endothelial exfoliation caused by various factors, the exposed vascular smooth muscle at the endothelium lesions migrate, undergo proliferation, and synthesize and secrete a large amount of ECM, which then leads to matrix deposition, vascular wall thickening, luminal stenosis, etc. Our data indicated that the epididymis tissue protein profilin-1 (PFN1) is differentially upregulated in rats fed a high-salt diet. PFN1 is known to inhibit the migration of certain cells, including T cells, by inhibiting the elongation of the branching ends of actin filaments (Schoppmeyer et al., 2017[29]). We hypothesize that it has a similar role in smooth muscle cells, that is, it plays a key role in inhibiting smooth muscle cell migration and vascular remodeling to maintain homeostasis due to the body's self-protection. Our KEGG signaling pathway analysis revealed that the identified DEPs coronin 1A (CORO1A), tubulin-α1A (TUBA1A), lysosomal associated membrane protein 1 (LAMP1), and THBS1 were all involved in processes involving phagosomes. Correct elimination of apoptotic cells is a necessary link for programmed cell death, organ development, and tissue homeostasis of an organism. Apoptotic cells lead to the maturation of phagosomes, and eventually the phagosomes that encapsulate the cell cadaver are fused with lysosomes to digest the phagosome contents (Pinto and Hengartner, 2012[26]. Protein AMBP was found in our LC-MS/MS results to be differentially increased in rats fed a high-salt diet. It is a matrix sugar complex that is involved in cell adhesion, vesicle-mediated transport, scavenger receptor binding, and ligand uptake, playing an important indirect role in organisms, such as in pathogen cleanup, lipid transport, antigen presentation, and apoptotic cell cleanup.

The top three fatal cardiovascular diseases worldwide, namely, myocardial infarction, stroke, and venous thromboembolism, have a common underlying "culprit": thrombosis. There are typically three major reasons for thrombosis: damage to the vascular endothelium, blood stasis, and a hypercoagulable state. A hypercoagulable state refers to the abnormality of certain components and coagulation function in the blood that causes the blood to easily coagulate into a thrombus. Fibrinogen-like protein 2 (FGL2; also known as Fibroleukin) is an immunosuppressant that cleaves prothrombin into thrombin, and then the fibrin alpha chain (FGA) polymerizes with fibrinogen beta chain and fibrinogen gamma chain to form insoluble fibrin, which directly triggers *in situ* thrombosis. In a study using an experimental model of pulmonary hypertension, *Fgl2* gene knockout significantly limited primary thrombosis (Fan et al., 2019[10]). In the present study, we found that FGL2 and FGA were significantly upregulated in the high-salt diet group. Given that FGL2 is also related to apoptosis, angiogenesis, and the inflammatory response, the upregulation of the fibrinolytic system associated with FGL2 and FGA may play an important role in the reduction of blood flow and increased vascular embolism induced by a high-salt diet. We also found that histidine-rich glycoprotein (HRG) is differentially upregulated in rats fed the high-salt diet. HRG contains two cystatin domains that are located in plasma and in platelets and is known to have two functions, namely, to inhibit fibrinolysis and to reduce the inhibitory effect on coagulation, indicating a potential thrombogenic effect. Our finding of a differential upregulation of FGL2 suggests that FGL2 with or without HRG may be a major risk factor for a hypercoagulable state induced by a high-salt diet and may be a potential drug target for clinical treatment of cardiovascular disease induced by a high-salt diet.

Numerous studies have shown that complement plays an important role in cardiovascular disease, and activation of the complement system can cause a variety of acute or chronic cardiovascular diseases or aggravate existing cardiovascular diseases to a large extent. In atherosclerosis, different proteins activate different complement pathways: C-Reactive Protein (CRP) bound to ligands, immune complexes, enzyme-modified low-density lipoproteins, apoptotic substances, and phospholipids. Mitochondrial proteins can activate classical complement pathways (Carter, 2012[5]). Complement C1q subcomponent subunit A (C1QA) chain is a polypeptide of the A1 chain of the serum complement subcomponent C1q in the classical pathway. This chain combines with C1r and C1s to produce the first component of the serum complement system. The differentially increased C1QA found in rats on a high-salt diet in our study provides further evidence that the classical pathway of complement activation is involved in cardiovascular damage due to high-salt diets by regulating key cascade responses and reducing complement activation; it is expected to become a new target for the treatment of cardiovascular disease. In addition, the complement system is also activated in patients with heart failure, which may be related to the direct activation of classical pathways and complement bypass pathways by necrotic or damaged spinous cells, or to the activation of complement by CRP in patient plasma (Frey et al., 2013[12]; Orrem et al., 2018[25]; Suffritti et al., 2017[33]). In the classical complement pathway pathogenesis of cardiovascular disease, CRP is typically expressed at high levels (Salazar et al., 2014[28]; Yasojima et al., 2001[39]), which is in contrast to our results. Whether the CRP-mediated inflammatory response is involved in the damage associated with a high-salt diet in cardiovascular disease remains to be further explored and confirmed.

Another protein that was differentially upregulated in rats fed a high-salt diet in the present study, and has also been detected in blood samples from patients with chronic heart failure (Wan et al., 2018[37]), was vascular non-inflammatory molecule 3 (VNN3). This finding suggests that the cardiovascular damage due to a high-salt diet may be associated with VNN3. Recently, it has been found that knockdown of lncRNAVNN3 decreases phosphorylation of Beclin-1 and apoptosis regulator Bcl-2 and mediates autophagy and apoptosis induced by 1,4-benzoquinone (Chen et al., 2019[6]). We hypothesized that VNN3 is involved in autophagy and apoptosis under conditions of high-salt consumption, suggesting that VNN3 may be a novel, early, sensitive biomarker for cardiovascular disease induced by a high-salt diet. Gem nuclear organelle associated protein 4 (GEMIN4) is a protein-coding gene, and diseases related to GEMIN4 include cataract, kidney deformities, and neurodevelopmental disorders of microcephaly. Some researchers have pointed out that GEMIN4 may be a serum biomarker of cardiovascular disease induced by air pollution (Ward-Caviness, 2019[38]). These findings suggest that GEMIN4 may also be involved in the adverse cardiovascular effects of a high-salt diet.

Among the differentially downregulated proteins found in the present study in rats fed a high-salt diet, group-specific component (Gc) is a plasma protein, but its functional role is not yet clear. The Gc protein is synthesized in the liver and is only known to bind vitamin D, vitamin D metabolites, and G actin. Clinical trials have shown that the concentration of the G actin-Gc complex in patients with liver necrosis is significantly lower than that of the control group (Lee et al., 1985[19]). Whether its downregulation associated with a high-salt diet in the present study influences other clinical outcomes remains to be explored. Apolipoprotein A1 (APOA1), a major protein in plasma, acts as a cofactor for lecithin cholesterol acyltransferase, participating in the reverse transport of cholesterol from tissues to the liver for excretion. The role of APOA1 as an anti-atherosclerotic agent is well known; therefore, its reduction observed in the present study should be recognized and considered important in individuals with a high-salt diet. Follistatin-related protein 1 (FSTL1), also found to be downregulated in the group with a high-salt diet, is thought to regulate certain growth factors associated with cell proliferation and differentiation. A mouse model of endocardial/endothelial cell FSTL1 deficiency has been reported to result in mitral valve deformation, leading to regurgitation, heart failure, and early cardiac death (Prakash et al., 2017[27]). Recent findings in studies of acute coronary syndrome and heart failure highlight the potential of FSTL1 as a therapeutic target for heart regeneration (Dong et al., 2015[7]). Therefore, identifying that FSTL1 is downregulated in rats fed a high-salt diet is of great value; however, further study is needed to determine the specific downstream growth factors that are influenced by the reduced FSTL1 and that may further affect the proliferation and differentiation of functional cells.

## Conclusions

The present study effectively used the iTRAQ technique for protein identification and quantification to discover changes in key proteins and their potential roles in the pathogenesis of cardiovascular diseases, such as hypertension, arteriosclerosis, and heart failure, associated with a high-salt diet. The identified proteins were related to the development of vascular endothelial dysfunction, vascular remodeling, oxidative stress, activation of the complement system, and imbalance in the fibrinolytic system. Our data indicated that the integrin-focal adhesion pathway and phagosome processes likely impact the pathology associated with a high-salt diet. Some of the key proteins identified in the present study may be considered as new therapeutic targets for high-salt intake-related cardiovascular diseases.

## Notes

Bing Shen and Jiexia Chen (Department of Geriatrics Cardiology, the First Affiliated Hospital of Anhui Medical University, 218 Jixi Road, Hefei, Anhui 230022, China; Tel: +86-551-65908416, E-mail: chenjiexia28@sina.com) contributed equally as corresponding authors.

## Funding

This work was supported by grants from the National Natural Science Foundation of China (grant Nos. 81570403, U1732157, and 8197102295); the Anhui Province Science and Technology Innovation Project Demonstration Project (No. 201707d08050003); and the Anhui Province Key Research and Development Project (No. 201904a07020032).

## Role of the funders

The funders had no role in study design; in the collection, analysis and interpretation of data; in the writing of the report; and in the decision to submit the article for publication.

## Declaration of competing interests

The authors declare that they have no conflicts of interest related to the contents of this article.

## Supplementary Material

Supplementary data

## Figures and Tables

**Table 1:**
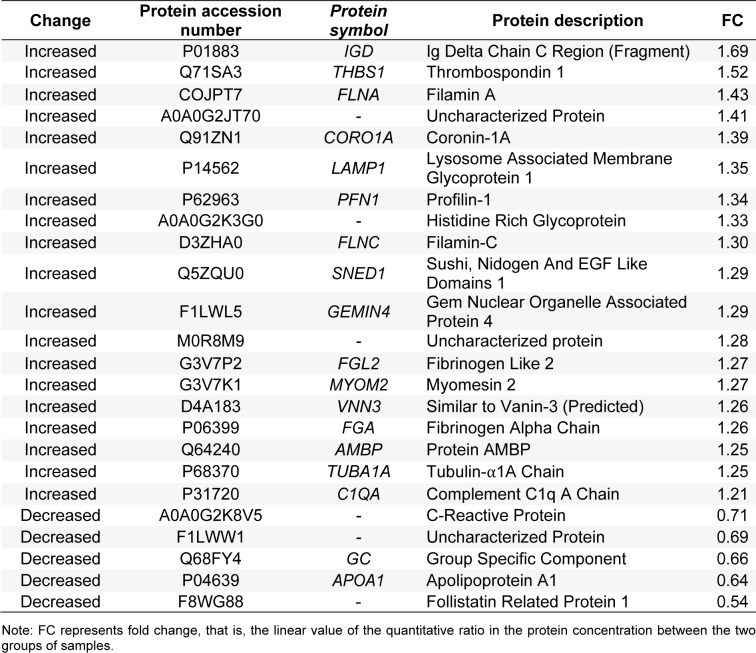
Differentially expressed serum proteins in rats fed a high-salt vs. regular diet
